# Detection of Rare Thalassemia Variants Using Accurate Circular Consensus Long‐Read Sequencing

**DOI:** 10.1155/humu/8897128

**Published:** 2026-01-11

**Authors:** Xiaoqiang Zhou, Yue Chen, Shufen Chen, Jingli Lian, Yue Liu, Tingting Yang, Shuijuan Wu, Juan Liu, Xiang Huang, Xingkun Yang

**Affiliations:** ^1^ Prenatal Diagnosis Center, The Affiliated Foshan Women and Children Hospital, Guangdong Medical University, Foshan City, Guangdong Province, China, gdmu.edu.cn; ^2^ Women and Children Medical Research Center, The Affiliated Foshan Women and Children Hospital, Guangdong Medical University, Foshan City, Guangdong Province, China, gdmu.edu.cn; ^3^ The Second School of Clinical Medicine, Southern Medical University, Guangzhou, Guangdong, China, fimmu.com

**Keywords:** accurate circular consensus long-read sequencing, diagnosis, thalassemia, variant

## Abstract

**Objective:**

The aim of this study is to evaluate the efficacy of accurate circular consensus long‐read sequencing in the detection of rare thalassemia.

**Methods:**

Conventional molecular analysis on globin genes has limitations because of the broad spectrum of genetic variants, complex genetics, and genotype–phenotype correlation. Accurate circular consensus long‐read sequencing is a novel tool that detects complex variants in the thalassemia gene based on third‐generation sequencing. In this study, we screen out suspected rare thalassemia carriers by hemoglobin analysis and conventional molecular analysis, and evaluate the efficacy of accurate circular consensus long‐read sequencing in the detection of rare thalassemia.

**Results:**

Based on the traditional screening of thalassemia gene, an additional 16 (17.67%) cases of clinically significant variants of rare thalassemia were identified by accurate circular consensus long‐read sequencing in this study, including 12‐point variants and 4 deletion variants: HBB: (SEA)‐HPFH, HBB: c.268_281delAGTGAGCTGCACTG, HBB: (Chinese) G*γ* + (A*γδβ*)0, and *HBA2*:c.91‐93delGAG.

**Conclusion:**

Accurate circular consensus long‐read sequencing has a promising prospect in detecting rare thalassemia gene variants and may improve the detection rate of carriers.

## 1. Introduction

Thalassemia is an autosomal recessive, common hereditary disease of the hematologic system that causes anemia worldwide. Its severity varies from asymptomatic, to mild microcytic and hypochromic anemia, and to severe anemia, especially in Southeast Asian, Middle Eastern, and Mediterranean populations. It has been estimated that approximately 1%–5% of the world population are carriers for a thalassemia‐causing variant, and it is listed as one of the hemoglobinopathies affecting global health by the World Health Organization [1–4]. In Guangdong and Guangxi provinces, which are located in Southern China, a country in Southeast Asia, a high carrier rate of 17.83% and 24.51% of thalassemia was estimated [5, 6]. The pathogenesis of thalassemia lies in the disturbance of the balance of globin chains, causing abnormal erythropoiesis, increased hemolysis, and anemia [7]. Clinical manifestations range from asymptomatic to lifelong transfusion treatment and even death [8, 9]. Despite the increasing awareness of thalassemia, many children are still born with severe thalassemia, which brings serious economic and psychological pressure to families.

*α*‐Thalassemia and *β*‐thalassemia are two main types of thalassemia, which are caused by variants of *HBA1* (OMIM 141800)/*HBA2* (OMIM 141850) and *HBB* (OMIM 141900) genes [10]. It is challenging to diagnose thalassemia carriers due to the complexity of genetics and genotype‐phenotype correlations. Common genetic tests for thalassemia include GAP‐PCR testing, reverse dot blot hybridization assays, multiple ligation‐dependent probe amplification (MLPA), Sanger sequencing, and next‐generation sequencing (NGS), but all of them have limitations in the detection of the thalassemia gene mutational spectrum [11]. GAP‐PCR and reverse dot blot hybridization assays can only detect specific variants that are already known. MLPA is usually used to detect deletion or duplication variants. Although Sanger sequencing is the gold standard for genetic testing, it can only identify point variants and indels. NGS has shortcomings in its capability to investigate complex genomes, repetitive elements, full‐length transcripts, or native base modifications, which may miss fusion genes and special variants [12–14]. Single‐molecule real‐time (SMRT) sequencing is a method based on third‐generation sequencing (TGS), which has the advantages of long‐read, high accuracy, single‐molecule resolution, and no GC bias. Moreover, circular consensus sequencing (CCS) can improve the accuracy of SMRT sequencing and generate highly accurate (99.8%) long high‐fidelity (HiFi) reads [15, 16]. It has been reported in recent years that TGS may be a novel tool for detecting multiple and complex variants, including large deletions or duplications, SNVs, indels, and fusion genes in the thalassemia gene, and a universal approach to comprehensive analysis of thalassemia alleles (CATSA) [17–20].

In Guangdong province, screening for thalassemia is routinely implemented in all pregnant women and their husbands by a blood test. Once low levels of MCV (< 80 fL) or MCH (< 27 pg), high or low levels of HbA_2_, or abnormal Hb variants are detected, we will detect the thalassemia gene using GAP‐PCR and the reverse dot blot hybridization assays. A total of 23 common variants are included in the test: *α*‐thalassemia deletions including *–*SEA (Southeast Asia): *−α* ^3.7^ and *−α* ^4.2^: *α*‐thalassemia point variants including Hb Constant Spring (Hb CS) (*HBA2*: c.427 T > C), Hb WS (*HBA2*: c.369C > G) and Hb Quong Sze (Hb QS) (*HBA2*: c.377 T > C): and 17 *β*‐thalassemia point variants [21]. Rare thalassemia variants are often missed, which may result in the birth of children with severe thalassemia when both spouses are carriers. Therefore, in this study, we use accurate circular consensus long‐read sequencing to detect rare thalassemia and hope that we can reduce the rate of missed diagnosis of thalassemia carriers.

## 2. Materials and Methods

### 2.1. Hematological Analysis

In Foshan Woman and Children Hospital, routine analysis of blood and hemoglobin electrophoresis [22] is performed on all pregnant women who undergo prenatal examinations at the obstetrics clinic and their husbands. We mainly collected suspected cases of thalassemia during the 2‐year period from 2021 to 2022. The inclusion criteria for patients were: either (1) routine analysis of blood showed low levels of MCV (< 80 fL) or MCH (< 27 pg), (2) hemoglobin electrophoresis showed high or low levels of HbA_2_, or (3) other abnormal Hb variants. The exclusion criterion for patients was asiderosis. Those who met the above criteria were suspected thalassemia carriers.

### 2.2. Traditional Molecular Analysis

GAP‐PCR testing and the reverse dot blot hybridization assays were performed in all the patients who were suspected of thalassemia by hematological analysis. GAP‐PCR tests large‐deletion *α*‐thalassemias including −SEA, *−α* ^3.7^ (rightward), and −*α* ^4.2^ (leftward) according to the manufacturer′s protocol. The reverse dot blot hybridization assays detect three common nondeletional *α*‐thalassemia variants including Hb CS (*HBA2*: c.427 T > C), Hb QS (*HBA2*: c.377 T > C), Hb Westmead (Hb WS) (*HBA2*: c.369C > G), and 17 *β*‐thalassemia point variants including codons 41/42 (−TCTT) (*HBB*: c.126‐129delTCTT), IVS‐II‐654 (C > T) (*HBB*: c.316‐197C > T), −28 (A > G) (*HBB*: c.‐78A > G), codons 71/72 (+A) (*HBB*: c.216_217insA), codon 17 (A > T) (*HBB*: c.52A > T), codon 26 (G > A) (Hb E) (*HBB*: c.79G > A), codon 31 (−C) (*HBB*: c.94delC), codons 27/28 (+C) (*HBB*: c.84_85insC), IVS‐I‐1 (G > T) (*HBB*: c.92+1G > T), codon 43 (G > T) (*HBB*:c.130G > T), −32 (C > A) (*HBB*: c.‐82C > A), initiation codon(ATG > AGG) (*HBB*: c.2 T > G), −30 (T > C) (*HBB*: c.‐80 T > C), codons 14/15 (+G) (*HBB*: c.45‐46insG), Cap +1 (A > C) (*HBB*: c.‐50A > C), −29 (A > G) (*HBB*: c.‐79A > G), and IVS‐I‐5 (G > C) (*HBB*: c.92+5G > C) (Yaneng Bioscience, Shenzhen, China). We will conduct CCS on patients suspected of rare thalassemia with abnormal results in routine analysis of blood or hemoglobin electrophoresis but tested negative by GAP‐PCR and the reverse dot blot hybridization assays.

### 2.3. Accurate Circular Consensus Long‐Read Sequencing and Date Analysis

Accurate circular consensus long‐read sequencing was performed as previously described [16, 23] in all patients who suspected thalassemia but tested negative by traditional molecular analysis or the phenotype did not match the detected genotype. Briefly, DNA extraction of the samples was performed using the QIAamp DNA Blood Mini Kit produced by Qiagen GMBH in Germany. Then, multiple long molecule PCR amplification was carried out, and a long fragment amplicon library was constructed. The DNA‐polymerase complex was loaded onto the SMRT chip, and sequencing was initiated on the PacBio Sequel II platform. CCS reads of the original subreads in BAM files output by the PacBio Sequel II sequencing platform were generated by CCS software (Pacific Biosciences). CCS reads were split, and barcode sequences were identified and clipped using Lima software in the Pbbioconda package (Pacific Biosciences). Then Split software (Pacific Biosciences) was used to split the samples and generate a separate BAM file. Subsequently, CCS reads filtered by cut barcode were compared with the human reference genome hg38 by pbmm2 (Pacific Biosciences) software to obtain the target gene fragment. FreeBayes1.3.4 software was then used to identify single‐nucleotide variations (SNV) and insertions and deletions (indels) for each haplotype. Reads length information was combined with HbVar, Ithanet, and LOVD databases to analyze variations larger than 50 bp. CCS reads of representative samples can be displayed and identified in an Integrative Genomics Viewer (IGV). The pathogenicity of the identified variants was classified according to the general guidelines and the hemoglobin variation database.

### 2.4. Conformation of Rare Variants Detected by CCS Using Other Methods

All SNVs detected by CCS were confirmed by Sanger sequencing. Based on the positive variant sites obtained through CCS, the PCR technology principle was applied for detection. Specifically, specific primers were designed at both ends of the mutant gene fragment to be tested for amplification. Then, agarose gel electrophoresis was used for analysis. The amplified products were subjected to Sanger sequencing to obtain the gene fragment sequence. Through Sanger sequencing, the mutations, short fragment deletions, or insertions at the positive sites were further analyzed and verified to determine the specific mutation situation of that site. Large‐deletion variants were confirmed by special PCR using primers spanning the known junction breakpoints.

## 3. Result

We collected 1617 suspected cases of thalassemia and diagnosed 1521 cases of thalassemia using traditional molecular analysis. Among them, 1034 are *α*‐thalassemia, 396 are *β*‐thalassemia, and 91 are both *α*‐thalassemia and *β*‐thalassemia. In *α*‐thalassemia, the most common mutation is −SEA, accounting for 51.91%, followed by −*α* ^3.7^, accounting for 23.29%. Among *β*‐thalassemia cases, the most common mutation is codons 41/42 (−TCTT), accounting for 36.96%. We totally collected 96 eligible peripheral blood samples for accurate circular consensus long‐read sequencing and identified 16 (17.67%) cases of clinically significant variants of rare thalassemia. The male‐to‐female ratio is 1:1. All the long‐read molecular sequencing data were verified by specific PCR and Sanger sequencing. The verification results are consistent with CCS. The results of hematological and genetic tests in 16 cases of rare thalassemia are shown in Table 1 and Table 2. We also found 17 cases of *HBD* gene mutations, which may result in a decrease in HbA_2_, with the most common variant being *HBD*: c.‐127 T > C, totaling 14 cases.

**Table 1:** The results of hematological analysis in patients with significant variants detected by CCS.

**Patients**	**Sex/years**	**Hb** **(g/l)**	**MCV** **(fL)**	**MCH** **(pg)**	**HbA**_**2**_ **(%)** ^**a**^	**HbF** **(%)**	**Genotype**	**Effect**
1	M/28	167	86.8	29.4	1.7	0	*HBA2*:c.80C > A	*α* ^+^‐thal
2	F/24	122	88.2	29.9	2.2	0	*HBA2*:c.91G > C	*α* ^+^‐thal
3	F/23	122	90.7	30	1.9	0	*HBA2*:c.34A > C	*α* ^+^‐thal
4	F/26	130	89.1	28.3	2	0	*HBA2*:c.80C > A	*α* ^+^‐thal
5	F/29	102	83	26.2	4.5^a^	39.25	*HBB*:(SEA)‐HPFH	*β* ^0^‐thal
6	M/42	148	71.4	24	8.41^a^	0	*HBB*:c.‐137C > T	*β* ^+^ or *β* ^++^‐thal
7	F/29	92	64.2	20.9	6.61^a^	0	*HBB*:c.316‐146 T > G	*β* ^+^‐thal
8	M/29	141	73.6	25	7.33^a^	0	*HBB*: c.‐136C > G	*β* ^+^‐thal
9	F/35	116	61.4	20.5	7.22^a^	0	*HBB*:c.268_281delAGTGAGCTGCACTG	*β* ^0^‐thal
10	M/2	103	60.8	20.2	7.14^a^	0	‐*α* ^4.2^ *HBB*:c.91A > G	*α* ^+^‐thal *β* ^0^‐thal
11	M/22	140	66.6	22.3	3.59^a^	0	*HBB*:(Chinese)G*γ* + (A*γδβ*)0	*β* ^0^‐thal
12	M/26	148	92.8	29.7	2.39^a^	0	*HBA2*:c.91G > C	*α* ^+^‐thal
13	F/35	87	70.7	20.9	3.11^a^	0	*HBA1*:c.34A > C	*α* ^+^‐thal
14	M/29	145	76.1	23.9	2.1	0	*HBA2*:c.91‐93delGAG	*α* ^+^‐thal
15	M/27	111	62.3	19.4	5.7	0	*HBB*:c.316‐146 T > G	*β* ^+^‐thal
16	F/38	182	91.3	31	1.7	0	*HBB*:‐50(G > A)	*β* ^+^‐thal

*Note:* Normal reference range of hemoglobin is 130–175 g/l in males and 115–150 g/l in females, MCV(82–100 fL), MCH(27–34 pg), HbF(0%–2%).

^a^The reference of HbA_2_ in Patient #5 to Patient #13 is 3.7%–6%, whereas others are 2.5%–3.5% because of laboratory change.

**Table 2:** Significant variants in *HBA1*, *HBA2*, and *HBB* gene detected by accurate circular consensus long‐read sequencing.

**Patients**	**Gap-PCR and reverse dot blot hybridization assays results**		**CCS result**		**Variant verifcation**
	***α*-thalassemia**	***β*-thalassemia**	***α*-thalassemia**	***β*-thalassemia**	
1	*αα*/*αα*	*N*	*HBA2*:c.80C > A	*N*	Sanger sequencing
2	*αα*/*αα*	*N*	*HBA2*:c.91G > C	*N*	Sanger sequencing
3	*αα*/*αα*	*N*	*HBA2*:c.34A > C	*N*	Sanger sequencing
4	*αα*/*αα*	*N*	*HBA2*:c.80C > A	*N*	Sanger sequencing
5	*αα*/*αα*	*N*	*N*	*HBB*:(SEA)‐HPFH	Electrophoresis
6	*αα*/*αα*	*N*	*N*	*HBB*:c.‐137C > T	Sanger sequencing
7	*αα*/*αα*	*N*	*N*	*HBB*:c.316‐146 T > G	Sanger sequencing
8	*αα*/*αα*	*N*	*N*	*HBB*: c.‐136C > G	Sanger sequencing
9	*αα*/*αα*	*N*	*N*	*HBB*:c.268_281delAGTGAGCTGCACTG	Sanger sequencing
10	−*α* ^4.2^/*αα*	*N*	−*α* ^4.2^	*HBB*:c.91A > G	Sanger sequencing
11	*αα*/*αα*	*N*	*N*	*HBB*:(Chinese)G*γ* + (A*γδβ*)0	Electrophoresis
12	*αα*/*αα*	*N*	*HBA2*:c.91G > C	*N*	Sanger sequencing
13	*αα*/*αα*	*N*	*HBA1*:c.34A > C	*N*	Sanger sequencing
14	*αα*/*αα*	*N*	*HBA2*:c.91‐93delGAG	*N*	Sanger sequencing
15	*αα*/*αα*	*N*	*N*	*HBB*:c.316‐146 T > G	Sanger sequencing
16	*αα*/*αα*	*N*	*N*	*HBB*:‐50(G > A)	Sanger sequencing

Based on the traditional detection methods, we identified an additional 12 cases of point mutations and 4 cases of deletion mutations: *HBB*: (SEA)‐HPFH, *HBB*:c.268_281delAGTGAGCTGCACTG, *HBB*:(Chinese)G*γ* + (A*γδβ*)0, and *HBA2*:c.91‐93delGAG. Among them, the heterozygous missense variants of *HBA2* all showed normal hemoglobin and low HbA_2_ levels, with or without decreased MCV and MCH. Anemia was present in five cases (Patients #5, #7, #10, #13, and #15), with only one male. Patient #10 showed low levels of Hb, MCH, and MCV, but a high level of HbA_2_. Only heterozygous −*α* ^4.2^ was identified by routine screening of thalassemia variants. In addition to the mutation −*α* ^4.2^ (Figure 1a) detected by traditional methods, we also identified a heterozygous variant (c.91A > G) in the *HBB* gene through CCS (Figure 1b). A double peak can be observed in the sequence verification graph of Sanger sequencing in Figure 1c, which indicates heterozygous mutation.

Figure 1Variants in Patients #10 detected by LRS (a) The pink area showed the −*α* ^4.2^ deletion, (b) the blue line in the pink area represents the *HBB* c.91A > G mutation, and (c) the sequence verification graph of Sanger sequencing. The arrow indicates the double peak.

**Figure:**
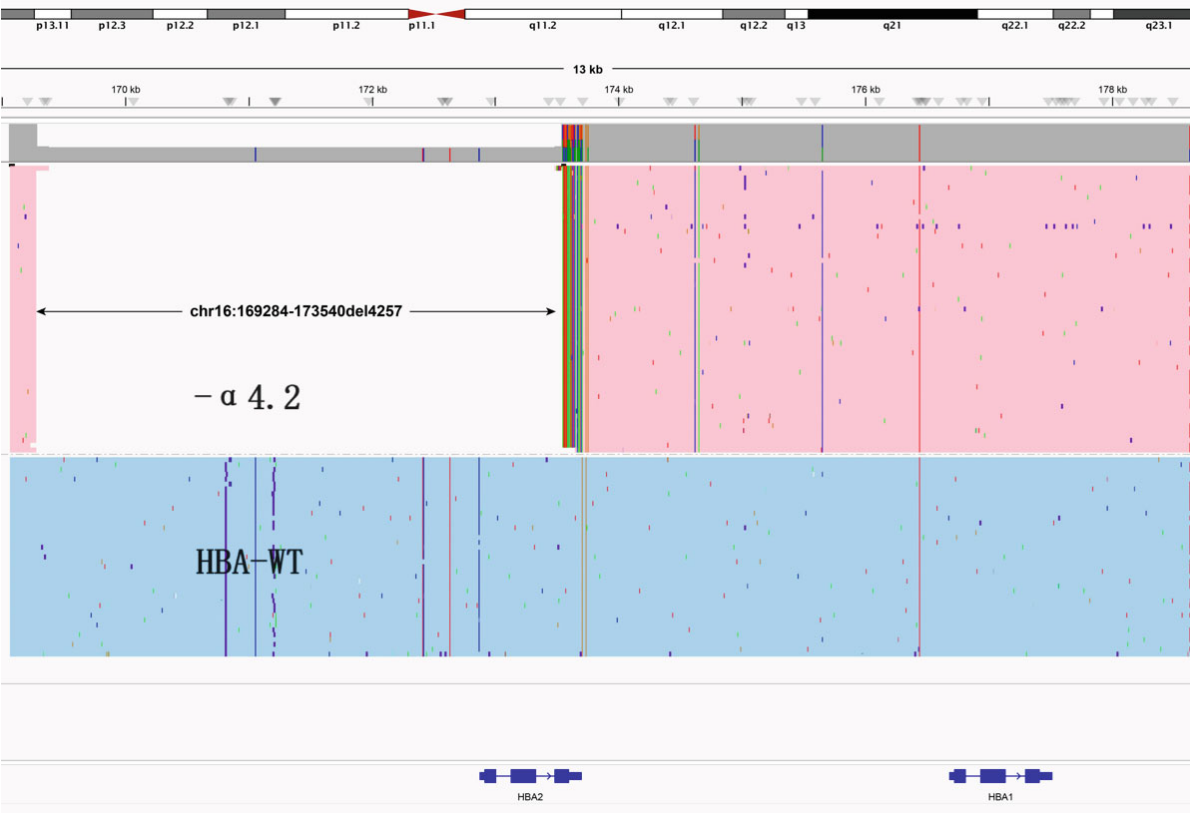
(a)

**Figure:**
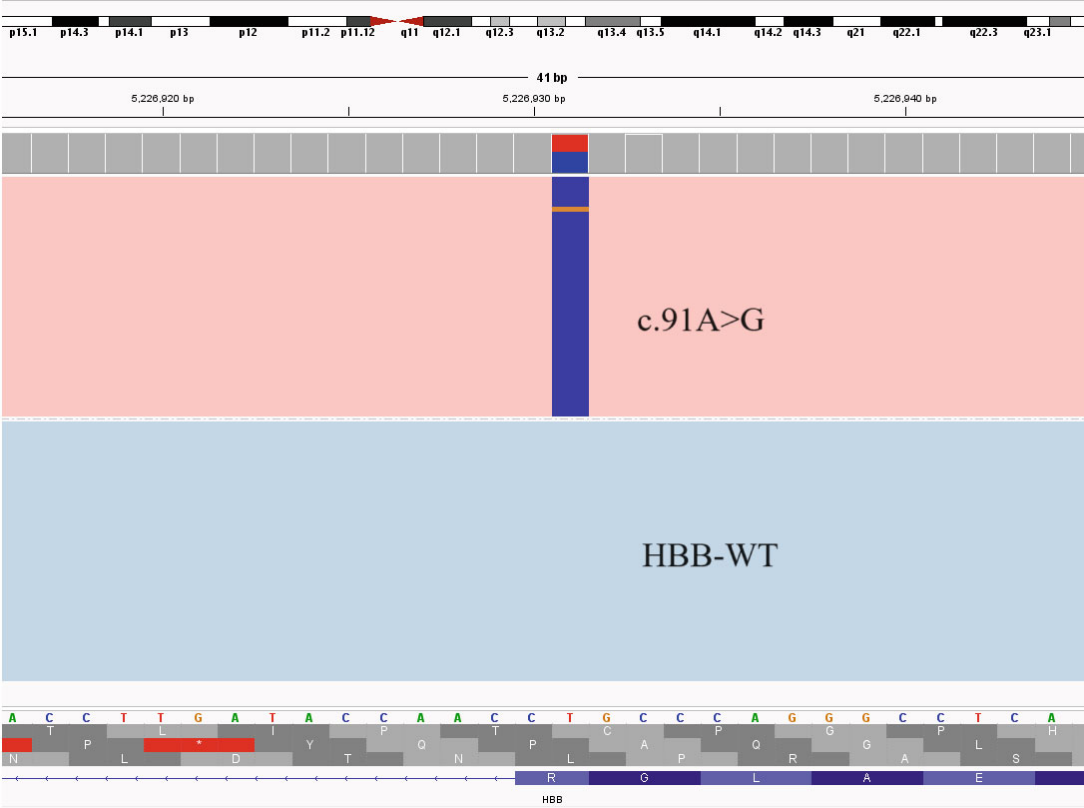
(b)

**Figure:**
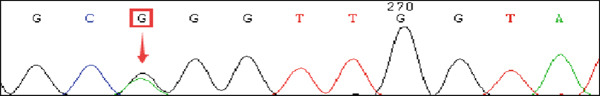
(c)

Two large‐deletion variants of HBB (Patient #5 and #11) were detected and verified by specific PCR (Figure 2), whereas common screening of thalassemia genes and Sanger sequencing could not identify them. We observed that the band amplified from Patient #5 is identical to that of the positive control *HBB*:(SEA)‐HPFH and Patient #11 is consistent with the positive control *HBB*:(Chinese)G*γ* + (A*γδβ*)0.

Figure 2Variants (a) HBB:(SEA)‐HPFH (A) in Patients #5 and (b) HBB:(Chinese)Gγ+(Aγδβ)0 (B) in Patients #11 identified by CCS and verified by (c,d) electrophoresis. (c,d) are the electrophoresis verification diagram.

**Figure:**
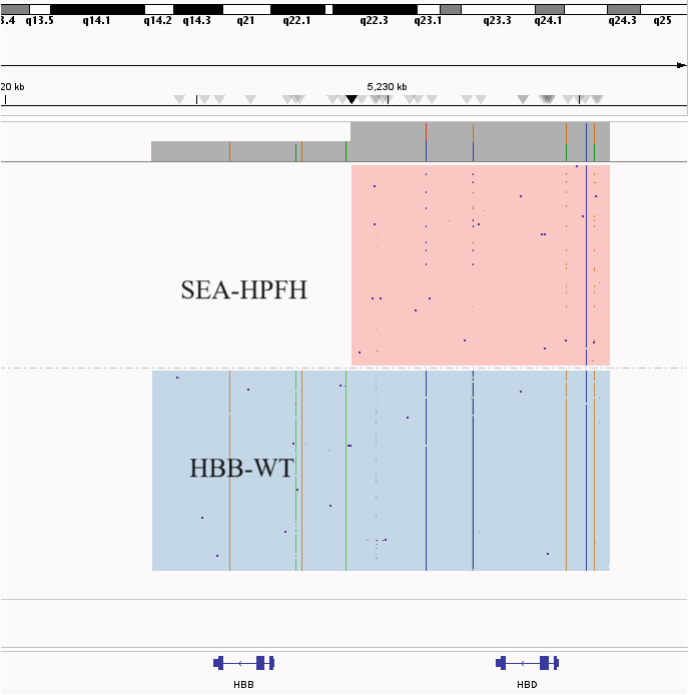
(a)

**Figure:**
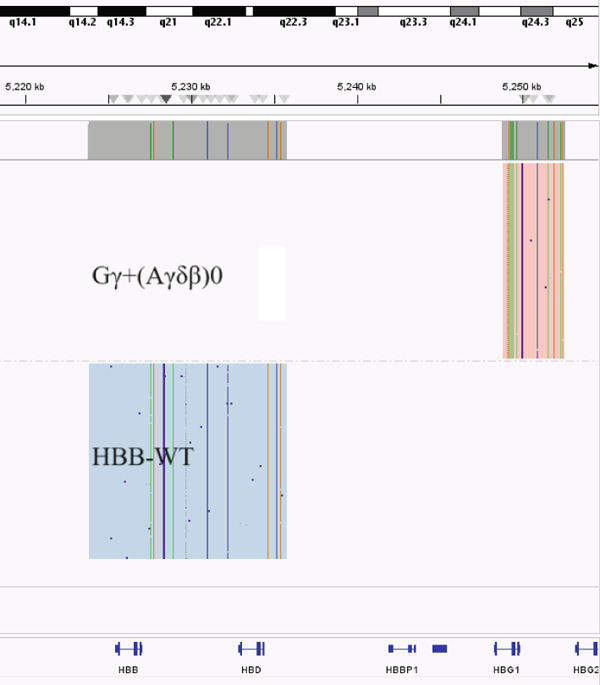
(b)

**Figure:**
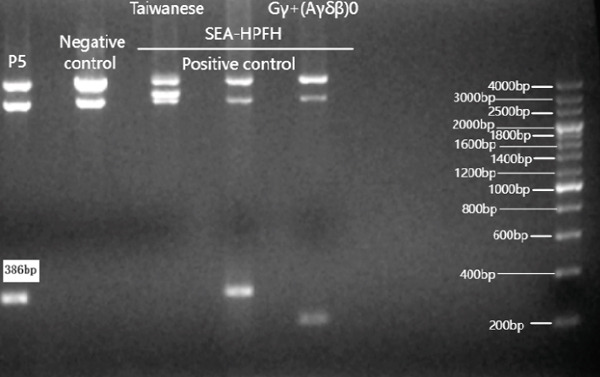
(c)

**Figure:**
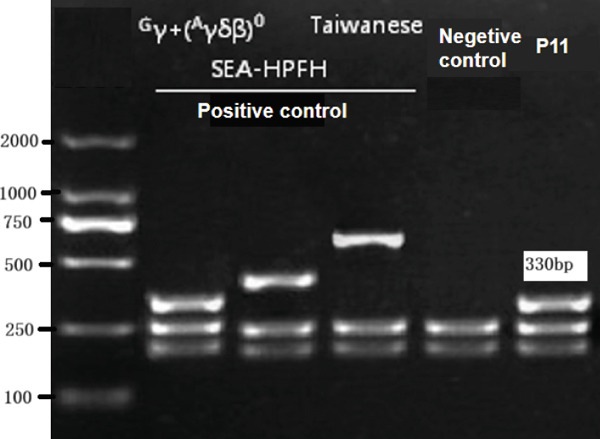
(d)

Patients #16 showed a high level of Hb, normal MCV and MCH, but low HbA_2_. We identified variant *HBB*:‐50(G > A) (Figure 3a), which could not explain the decrease of HbA_2_ because *β*‐thalassemia alone usually results in an elevation of HbA_2_. Therefore, we detected the *δ*‐globin gene by Sanger sequencing, which may cause low HbA_2_ but normal MCV and MCH, and we found the variant codon 106(TTG‐>TCG)(Leu‐>Ser) in delta (Figure 3b).

Figure 3Variants in Patients #16. We identified variants (a) *HBB*:‐50(G > A) through CCS and (b) codon 106(TTG‐>TCG)(Leu‐>Ser) in delta by Sanger sequence.

**Figure:**
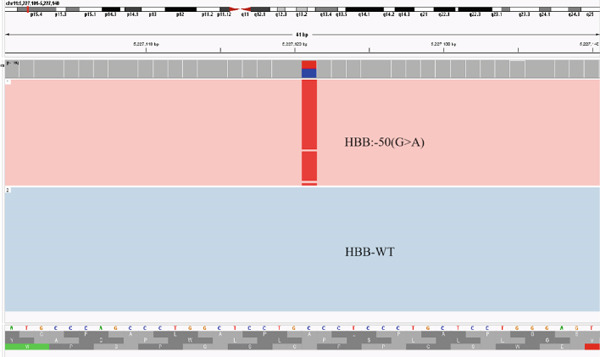
(a)

**Figure:**
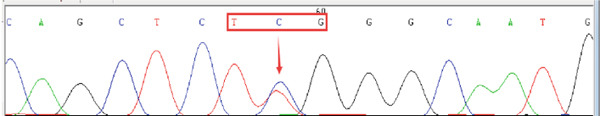
(b)

**Figure 4:**
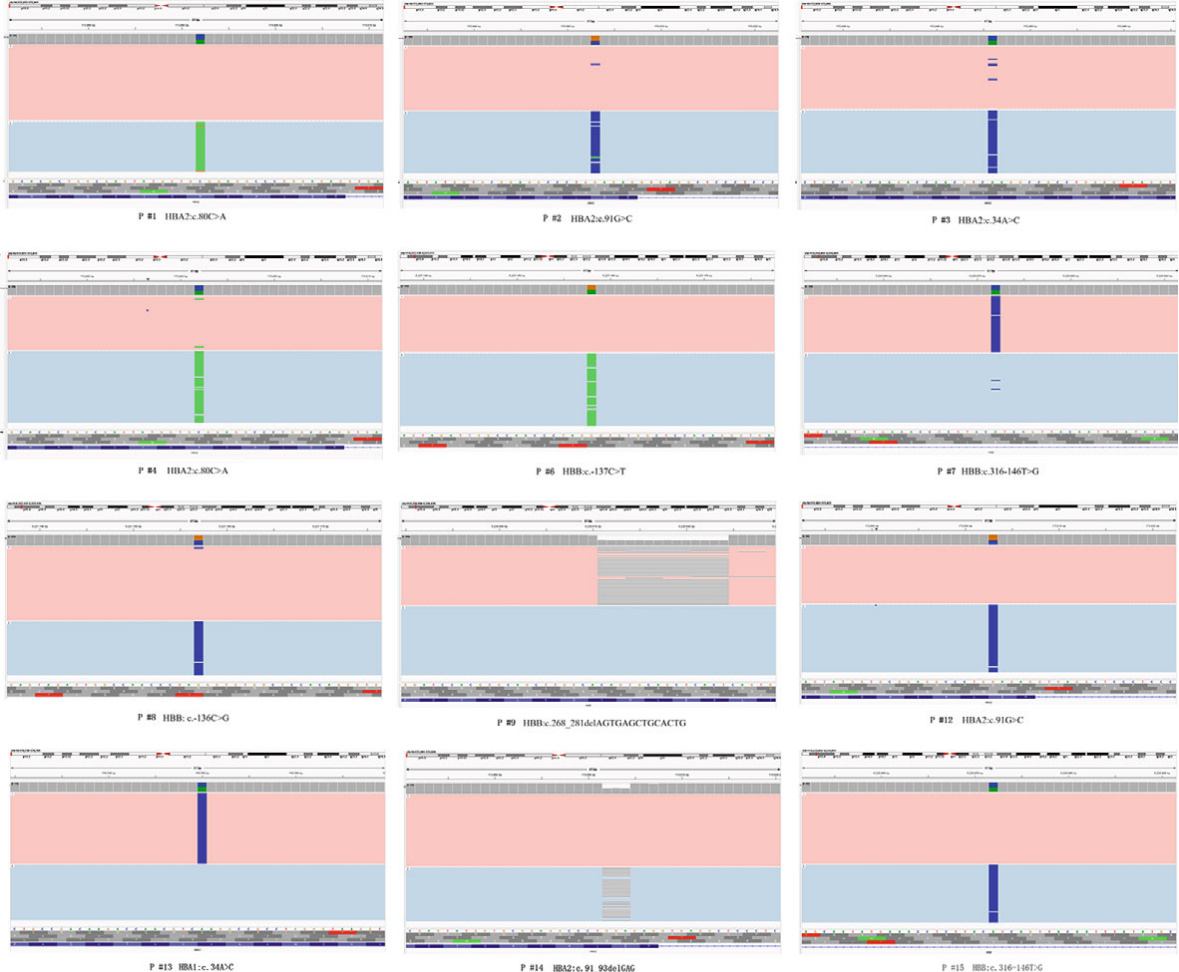
Identification of other clinically significant SNVs and indels by CCS. The graphs are the results exported from Integrative Genomics Viewer (IGV).

We identified additional clinically significant SNVs and indel variants through CCS analysis (Figure 4).

## 4. Disscussion

Thalassemia is a very common genetic disorder in Southern China, especially in Guangdong and Guangxi provinces. The high incidence of thalassemia compels us to perform prenatal screening, but because about 2000 variants of human hemoglobin variants and thalassemia were included in the HbVar database (https://globin.bx.psu.edu/globin/hbvar/menu.html), the traditional methods such as GAP‐PCR, Sanger sequencing, reverse dot blot hybridization assays, and so on cannot meet our needs. The types of variants in thalassemia that have been reported include SNVs, indels, large fragment deletions and duplications, gene fusions, triplets, and so on in the HbVar database. Sanger sequencing can only detect SNVs and indels and cannot recognize complex and large variants. GAP‐PCR and reverse dot blot hybridization assays can identify specific known variants, and GAP‐PCR may misdiagnose Hong Kong *αα* as −*α* ^3.7^ [24, 25]. The limited range of traditional genetic tests for thalassemia may lead to missed diagnosis of thalassemia carriers, resulting in the birth of children with severe thalassemia, which brings various pressures to families, especially in areas with high incidence of thalassemia, or result in misdiagnosis and increased anxiety in pregnant women.

In this study, 96 eligible samples were detected, but only 16 (17.67%) cases of clinically significant variants of rare thalassaemia were identified by CCS. Possible causes include the presence of other hemoglobinopathies such as variants in the *δ*‐globin gene that cause a decrease in HbA_2_ or siderosis, but serum iron was in the normal range due to iron supplementation. Indeed, we detected other variations in the *HBD* gene that may cause abnormal HbA_2_. Other possible causes are similar manifestations of thalassemia caused by variants in the regulatory elements of the *α*‐thalassemia or *β*‐thalassemia genes, as well as genetic modifiers of thalassemia, such as HS40 deletion of the *α*‐thalassemia regulatory region, which can lead to mild manifestations of thalassemia [26, 27]. In a study conducted in 2022, Cuiting Peng and his team tested 100 blood samples suspected of rare thalassemia using the PacBio Sequel II platform, and used electrophoresis and Sanger sequencing for verification. The results showed that TGS detected 10 rare thalassemia mutations and had a detection accuracy rate of up to 100% [28]. The rate of additional tests in our study was slightly higher than this, which might be because our research excluded some samples with abnormal serum iron levels.

Anemia was present in five (5/16) cases (Patients #5, #7, #10, #13, and #15) with only one male, even though the male‐to‐female ratio is 1:1. It may be related to the fact that the women were pregnant, and pregnant women are prone to anemia due to increased fetal consumption. Six (6/16) cases (Patients #1, #2, #3, #4, #12, and #16) had normal Hb, MCV, MCH by blood routine examination, which detected significant variants. Except for Patient #13, we observed that almost all patients solely characterized by the presence of *α* ^+^ showed no signs of anemia, indicating that *α* ^+^ is rarely associated with anemia. It suggests that using only blood routine screening for thalassemia may miss the diagnosis of mild thalassemia carriers like *α*+. Therefore, if blood counts of both spouses are normal but they have positive hemoglobin results, and if either one or both have a negative result from routine thalassemia gene testing, it is recommended that the negative individuals undergo third‐generation thalassemia testing to avoid missing the diagnosis of severely affected children with thalassemia. Patient #10 showed low levels of Hb, MCH, and MCV, but a high level of HbA_2_. Only heterozygous −*α* ^4.2^ was identified by routine screening of thalassemia deletions or variants, which cannot explain the rise of HbA_2_. We further identified a heterozygous variant in the *HBB* gene (c.91A>G) by the CCS in this patient, and avoid the missed diagnosis of *β*‐thalassaemia. It means a high level of HbA_2_ may be tested in patients who have both *α*+ and *β* ^0^. We observed that Patient #10 presented with mild anemia, despite also suffering from both alpha and beta types of thalassemia. This is because the pathogenesis of thalassemia lies in the disturbance of the balance of alpha and beta globin chains. When both the *α* and *β* chains decrease simultaneously, the symptoms are milder than those of a single type of thalassemia. The remaining nine cases (Patients #5, #6, #7, #8, #9, #11, #13, #14, and #15) have significant hematological abnormalities, but test negative using traditional methods. We finally found significant variants in them, which were confirmed by Sanger sequencing and electrophoresis. This may prevent the birth of children with moderate–severe *β*‐thalassemia. Therefore, when the results of hematology and traditional thalassemia gene tests cannot match, we should consider testing for rare thalassemia variants to avoid misdiagnosis of thalassemia carriers.

Accurate circular consensus long‐read sequencing (CCS) is one of the sequencing modes of TGS and has been reported with many advantages in genetic testing of thalassemia because of its long‐read, high accuracy, single‐molecule resolution, and lack of GC bias. First, it can distinguish the two homologous *α*‐globin genes, *HBA2* and *HBA1*, and a wide spectrum of variants can be detected. Second, cis–trans relationships between two or more variant sites can be recognized. Third, it makes structure variants clear, allowing the identification of specific variants such as −*α* ^3.7III^, *ααα* ^anti3.7^ and *ααα* ^anti4.2^, which can not be found by traditional methods [28–30]. Finally, the cost can be greatly reduced by only targeting the thalassemia gene region. Currently, the cost of CCS for thalassemia testing is higher than that of traditional methods; therefore, it can serve as a supplementary method for traditional methods. With the continuous reduction in the cost of TGS technology, it is expected to become the first‐line technology for clinical thalassemia gene screening in the future. In this study, all the clinically significant variants were confirmed by special PCR or Sanger sequencing, which verified the accuracy of the CCS. Although the original error rate of TGS is relatively high (about 10%–15%), mainly because it is real‐time sequencing and the complexity of signal interpretation is higher, CCS can reduce the false positive rate of the final results to below 0.1% by increasing the sequencing depth. Therefore, CCS has absolute advantages in the diagnosis of thalassemia carriers, especially in rare thalassemia, which can reduce the missed diagnosis of moderate or severe thalassemia fetuses before birth. It is an important component in the prevention and control of birth defects. One concern about the findings was that the study only used a small sample size (96 cases), and only samples from the Foshan area were included. Future research should conduct multicenter, large‐sample studies to verify the reliability of the results.

## 5. Conclusion

In conclusion, we identified 16 additional cases of clinically significant variants of thalassemia through accurate circular consensus long‐read sequencing compared with traditional methods from 96 cases with suspected thalassemia in this study. The variants include SNVs, indels, and large deletions. Our approach significantly reduces false‐negative results and misdiagnoses in thalassemia carriers. Combined with the results of three‐generation sequencing of thalassemia genes reported in previous literature, we believe that accurate circular consensus long‐read sequencing has a promising prospect in the detection of thalassemia genes, especially in rare variants. Therefore, we believe that TGS technology is expected to be applied to the detection of suspected samples that yield negative results in traditional clinical tests.

## Ethics Statement

The studies involving human participants were reviewed and approved by the Medical Ethics Committee of Foshan Women and Children Hospital. ID for ethics approval is FSFY‐MEC‐2024‐067. Investigations were conducted according to the Declaration of Helsinki. Informed consent was obtained from the individual(s) for the publication of any potentially identifiable images or data included in this article.

## Disclosure

All authors commented on previous versions of the manuscript. All authors read and approved the final manuscript.

## Conflicts of Interest

The authors declare no conflict of interest.

## Author Contributions

Xiaoqiang Zhou and Yue Chen contributed equally to this work. Xiaoqiang Zhou wrote the first draft of the main manuscript. Yue Chen wrote the final draft of the main manuscript. Shufen Chen did the traditional testing of thalassemia and verified the significant variants. The figures and tables were performed by Jingli Lian. Material preparation and data collection were performed by Yue Liu, Tingting Yang, and Shuijuan Wu. Data analysis was performed by Juan Liu. Xiang Huang and Xingkun Yang reviewed the manuscript. Xiaoqiang Zhou and Yue Chen contributed equally to this work and share first authorship.

## Funding

This study was supported by the Foshan Science and Technology Bureau (2320001006902) and the National Natural Science Foundation of China (82171713).

## Data Availability

The datasets generated and/or analysed during the current study are not publicly available due to ethics or patient privacy, but are available from the corresponding authors on reasonable request.
